# IQGAP1 Is a Scaffold of the Core Proteins of the Hippo Pathway and Negatively Regulates the Pro-Apoptotic Signal Mediated by This Pathway

**DOI:** 10.3390/cells10020478

**Published:** 2021-02-23

**Authors:** Niall P. Quinn, Lucía García-Gutiérrez, Carolanne Doherty, Alexander von Kriegsheim, Emma Fallahi, David B. Sacks, David Matallanas

**Affiliations:** 1 Systems Biology Ireland, School of Medicine, University College Dublin, Belfield, Dublin 4, Ireland; niall.quinn1@ucd.ie (N.P.Q.); lucia.garcia@ucd.ie (L.G.-G.); carolanne2.doherty@gmail.com (C.D.); emma.fallahi@gmail.com (E.F.); 2 Cancer Research UK Edinburgh Centre, Institute of Genetics and Molecular Medicine, University of Edinburgh, Edinburgh EH4 2XR, UK; Alex.VonKriegsheim@igmm.ed.ac.uk; 3 Department of Laboratory Medicine, National Institutes of Health, 10 Center Drive, Bethesda, MD 20892, USA; david.sacks2@nih.gov

**Keywords:** IQGAP1, MST2, LATS1, YAP1, Hippo, bile acid, hepatocellular carcinoma

## Abstract

The Hippo pathway regulates a complex signalling network which mediates several biological functions including cell proliferation, organ size and apoptosis. Several scaffold proteins regulate the crosstalk of the members of the pathway with other signalling pathways and play an important role in the diverse output controlled by this pathway. In this study we have identified the scaffold protein IQGAP1 as a novel interactor of the core kinases of the Hippo pathway, MST2 and LATS1. Our results indicate that IQGAP1 scaffolds MST2 and LATS1 supresses their kinase activity and YAP1-dependent transcription. Additionally, we show that IQGAP1 is a negative regulator of the non-canonical pro-apoptotic pathway and may enable the crosstalk between this pathway and the ERK and AKT signalling modules. Our data also show that bile acids regulate the IQGAP1-MST2-LATS1 signalling module in hepatocellular carcinoma cells, which could be necessary for the inhibition of MST2-dependent apoptosis and hepatocyte transformation.

## 1. Introduction

The Hippo pathway is emerging as a key regulator of different cell fates, such as proliferation, cell differentiation and apoptosis [1]. The core proteins of this pathway are conserved through evolution and include the kinase cassette formed by MST1/2 and LATS1/2 kinases and the co-transcriptional co-activator YAP1 [2,3]. Intensive work in the last decade has demonstrated that this pathway is part of a complex signalling network [1]. These data demonstrate that the core proteins of the Hippo pathway regulate cell fate by crosstalk with other signalling pathways, such as MAPK, AKT and WNT. The topology of this network is still poorly characterised and the mechanisms that mediate these crosstalks are not fully understood [4].

We and others have shown that the signal mediated by the core proteins of the pathway are regulated by several scaffold proteins [2,5,6,7]. The best-characterised scaffolds of the pathway are Salvador (Sav) and the RASSF family proteins (RASSF1-6) [1,2,8]. Sav is considered part of the canonical Hippo pathway, binds to and activates MST2 and LATS1 and promotes YAP1-S127 phosphorylation and cytoplasmic localisation [2]. Inhibition of the core kinases results in YAP1 translocation to the nucleus, where it binds the transcription factor TEAD and mediates the activation of the pro-survival transcriptional program. On the other hand, the role of the RASSF family seems to be more complex, as these proteins induce different cell fates [8,9]. The tumour suppressor RASSF1A, one of the most commonly deregulated genes in cancer, also scaffolds the interaction of MST2 and LATS1 and promotes the activation of these kinases [5,10,11]. Subsequently, activated LATS1 phosphorylates YAP1 and promotes its translocation to the nucleus. In this case, nuclear YAP1 interacts with p73 and the complex promotes the transcription of pro-apoptotic genes such as PUMA, which ultimately results in the initiation of apoptosis [1,5,12,13]. Other scaffold and adaptor proteins have been described to be part of this signalling network including KIBRA, DLG5, AMOT and the MOB family of proteins [14,15,16]. Thus, regulation of the Hippo pathway by protein–protein interactions, and by scaffolds in particular, are emerging as key mechanisms of this network. Scaffolds directly determine the diverse biological outcomes mediated by this pathway and the crosstalk of the core proteins with other pathways.

The IQGAP1-3 family of scaffold proteins are regulators of different biological processes, such as migration, proliferation, cytoskeleton regulation and cell to cell contact [17]. These classical scaffolds mediate their biological functions by binding key nodes of signalling pathways including RAF, MEK and ERK protein families, Rac1 and Cdc42, E-cadherin and β-catenin and are considered important regulators of intracellular dynamic signalling and protein localisation [18,19]. While IQGAP2 and -3 expression is restricted to specific organs, IQGAP1 is ubiquitously expressed and has been shown to be deregulated in different tumour types such as liver cancer, where it behaves as a putative oncogene [18,19]. Importantly, this scaffold protein also binds to YAP1, and in doing so, regulates the physiological functions of YAP1 by preventing YAP1-TEAD-dependent transcription [20]. Moreover, an involvement of IQGAP1 in YAP1-driven oncogenesis was proposed in liver cancer [21]. This work showed that IQGAP1 effects on YAP1-signalling were shown to be mediated by bile acids, which are arising as important regulators of Hippo signally downstream of FGFR4 [22,23,24] 

Here, we identify IQGAP1 as a new regulator of the core kinases of the MST2 pathway and confirm that it regulates YAP1 signalling. In particular, we characterise the molecular mechanistic effect of IQGAP1 in the Hippo pathway and show that this protein prevents the activation of the pro-apoptotic signal mediated by the pathway. Our data also confirm that IQGAP1 also regulates the pro-survival signal mediated by TEAD. Finally, we show data that indicate that IQGAP1 might be facilitating the crosstalk of the ERK1-AKT-Hippo network. 

## 2. Materials and Methods

### 2.1. Constructs and siRNA

Constructs encoding Myc-IQGAP1 (WT), IQGAP1ΔWW (ΔWW), IQGAP1ΔIQ (ΔIQ), IQGAP1ΔCHD (ΔCHD), IQGAP1-N (N), IQGAP1-N1 (N1) and IQGAP1-N2 (N2) have been described before [25]. HA-RASSF1A, FLAG-MST2, FLAG-LATS1 Myc-LATS1 (D846A), Flag-YAP1, Flag-YAP1 (S127A), HA-p73 and β-Galactosidase, have been described before [5,10,25,26,27]. p73 luciferase reporter PUMA-Frag1 (a gift from Bert Vogelstein, Addgene plasmid # 16591) and luciferase reporter TEAD 8XGTIIC LUC (a gift from Stefano Piccolo, Addgene plasmid # 34615) constructs were acquired from Addgene (Watertown, MA, USA ) [28]. Small interfering RNAs (siRNAs) against MST2, LATS1 and YAP1 were from Dharmacon (Lafayette, CO, USA) and have been validated before [5]; IQGAP1 siRNA LQ-004694-00- was also from Dharmancon.

### 2.2. Cell Culture and Transfections

Cells were grown in Dulbecco’s modified Eagle’s medium supplemented with 10% foetal calf serum. HeLa and HEK-293 cells were transfected using Lipofectamine 2000 (Invitrogen, Carlsbad CA, USA) following manufacturer’s instructions. HepG2 sub-confluent cells were transfected with Transit-X2^®^ Dynamic Delivery System (Mirus, Madison, WI, USA abbr. if USA, country) by following the manufacturer’s instructions.

### 2.3. Immunoprecipitation and Immunoblotting

Immunoprecipitations were performed as described before [5]. Briefly, cells were lysed in 20 mM HEPES, pH 7.5, 150 mM NaCl, 1% NP-40, 2 mM NaF, 10 mM -glycerophosphate, 2 mM Na_4_P_2_O_4_ and protease and phosphatase inhibitors. Lysates were cleared of debris by centrifugation at 14,000 RPM for 5 min. For indicated immunoprecipitations, cell lysates were divided in half and two immunoprecipitations were performed using specific antibodies. After incubation at 4 °C for 2 h, immunoprecipitates were washed three times with lysis buffer containing 0.5% NP-40, separated by SDS-PAGE, and analysed by western blotting. Where indicated, blots were quantified by densitometry using ImageJ [29].

### 2.4. Antibodies

All antibodies were from commercial sources: HA—horseradish peroxidase (anti-HA-HRP), 3F10 (Roche, Basel, Switzerland), rabbit monoclonal anti-MST2 (Abcam), goat polyclonal anti-MST2 (C-19; Santa Cruz, CA, USA), rabbit polyclonal p-T180-MST2 (Cell Signaling, Danvers, MA, USA), goat polyclonal anti-LATS1 (*n*-18 and g-16; Santa Cruz), rabbit monoclonal p-T1079-LATS1, rabbit polyclonal anti-YAP1 (Santa Cruz), mouse monoclonal anti-YAP1 (Sigma, Dallas, TX, USA), rabbit polyclonal p-S127-YAP (Cell Signaling), mouse monoclonal anti-IQGAP1 (MBL), rabbit polyclonal anti-IQGAP1 (Santa Cruz), mouse monoclonal C-Myc tag (Santa Cruz), AKT, p-S308-AKT, p-S473-AKT (Cell Signaling), mouse monoclonal anti-Tubulin and rabbit monoclonal anti-GAPDH (Santa Cruz), mouse monoclonal anti-FLAG M2-Peroxidase (Sigma), rabbit monoclonal anti-GFP, mouse monoclonal anti-TEF-1 (BD Biosciences), mouse monoclonal anti-p-T183/Y185-ERK1/2 and rabbit polyclonal anti-ERK1/2 (Sigma). Mouse monoclonal anti-GFP (Roche), rabbit anti-GFP or goat antiserum (Sigma-Aldrich, St Louis, MO, USA) were used as isotopic IgG immunoprecipitation control.

### 2.5. Luciferase Reporter Assays

Cells were seeded in six-well plates and transiently co-transfected with a plasmid coding for a Firefly-derived luciferase under the control of PUMA Frag1-Luc luciferase reporter [28] or TEAD luciferase reporter [30] and β-galactosidase under the control of an SV40 promoter. Cells were lysed using a Luciferase Reporter Assay System Kit (Promega) following manufacturer’s instructions. Luciferase luminescent was read at 130 nm in a SpectraMax microplate reader. β-galactosidase activity was measured using an assay mix (100 mM sodium phosphate, pH 7.0; 1 mM MgCl2; 50 mM β-mercaptoethanol; and 0.665 mg/mL ONPG in distilled water). The reaction was stopped using Na_2_CO_3_ and β-galactosidase activity was measured by reading absorbance at 450 nm on the microplate reader. Experiments using Firefly-derived luciferase were normalised to the activity of co-transfected β-galactosidase.

### 2.6. Real Time PCR (rtPCR)

RNA extraction was carried out using RNeasy^®^ Plus Mini Kit (QIAGEN, Manchester, UK) according to manufacturer’s protocol. For cDNA conversion, the SensiFAST TM cDNA Synthesis Kit (Bioline, London, UK) was used following the manufacturer’s protocol for 1 µg of RNA as template. SensiFAST TM SYBR (Bioline) was used to amplify cDNA in a QuantStudio (TM) 7 Flex System from Applied Biosystems. Gene expression was normalised against GAPDH levels. Primers (5′-3′): CTGF, Fw: TGTGTGACGAGCCCAAGGA, Rv: TCTGGGCCAAACGTGTCTTC; PUMA, Fw: CCTGGAGGGTCCTGTACAATCT, Rv: GCACCTAATTGGGCTCCATCT; GAPDH, Fw: GAGTCAACGGATTTGGTCGT, Rv: TTGATTTTGGAGGGATCTCG.

### 2.7. Cell Cycle and Apoptosis Assays

Cells were transfected and where indicated they were serum deprived for 16 h. Cells were trypsinised and divided for use into two separated experiments. Cell cycle and cell death levels were measured by assessing DNA fragmentation using PI staining (Sigma) by fluorescence-activated cell sorter (FACS) as described before [5]. The graphs show the quantitation of cells with fragmented (i.e., sub-G1, G1, S-phase and G2) DNA content from at least three independent experiments. Error bars represent standard deviations. The caspase assay (CaspACE™ FITC-VAD-FMK) was used to measure apoptosis, which assesses caspase activity using an in situ marker, a fluorescent analogue of the pan caspase inhibitor Z-VAD-FMK (Promega), as previously described [5]. After incubation, samples were analysed by flow cytometry (BD ACCURI C6).

## 3. Results

### 3.1. IQGAP1 Specifically Interact with the Core Proteins of the Hippo Pathway

**Figure 1:**
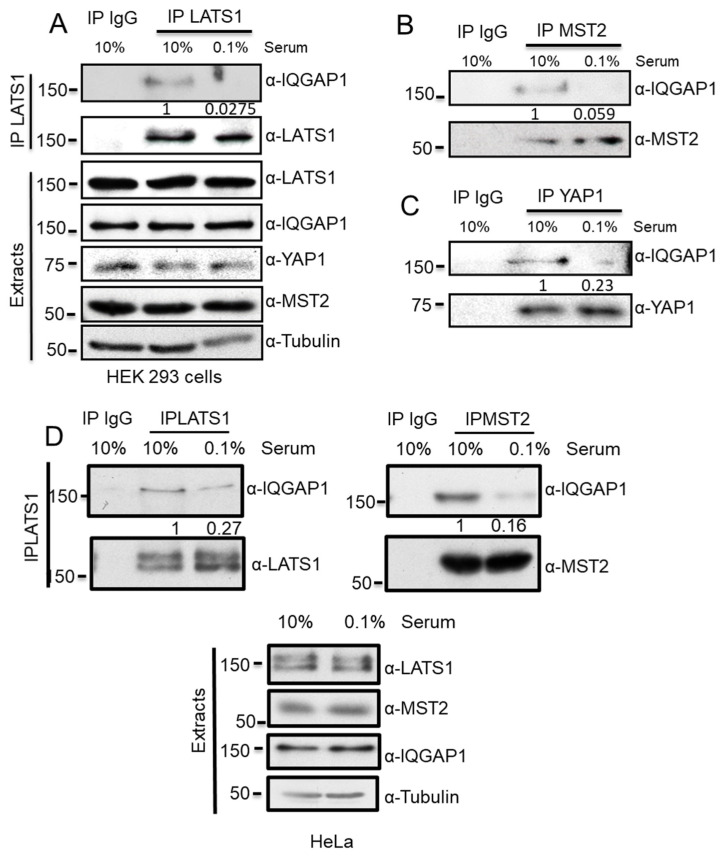
IQGAP specifically interacts with the core proteins of the MST/Hippo pathway. HEK 293 cells were grown in 10% serum or serum deprived (0.1% serum) for 16 h. (**A**) Endogenous LATS1 immunoprecipitates were analysed by western blot with the indicated antibodies. (**B**) Endogenous MST2 immunoprecipitates were analysed by western blot with the indicated antibodies. (**C**) Endogenous YAP1 immunoprecipitates were analysed by western blot with the indicated antibodies. IgG indicates isotypic antibody that was used as negative control for unspecific binding to protein G agarose beads. (**D**) HeLa cells grown in 10% serum or 0.1% serum and endogenous LATS1 or MST2 were immunoprecipitated. IQGAP co-immunoprecipitation was monitored using a specific antibody. Expression of the indicated proteins in cell extracts was detected by blotting with the indicated antibodies. IP blots were quantified using ImageJ and the numbers show relative fold change of IQGAP1 normalised by LATS1, MST2 or YAP1 IP blots, as indicated.

The core proteins of the Hippo pathway, MST2, LATS1 and YAP, can regulate different biological functions. How distinct cell fates are regulated by the same machinery is not yet fully understood. For this reason, we decided to mine a series of published and unpublished interaction proteomics screens with the aim to identify uncharacterised regulators and scaffolds of this pathway [10,31,32]. These experiments were performed by overexpressing GFP-LATS1, FLAG-LATS1, GFP-MST2 and GFP-YAP and FLAG-YAP1 in HEK-293 and C2C12 muscle cells grown in the presence or absence of serum. One protein that was identified as a putative interactor of the core proteins of the Hippo pathway in these datasets was IQGAP1, suggesting that this protein might be a regulator of this signalling network. Importantly, work from Sacks’s group have already identified YAP1 as an IQGAP1 interactor [20], which led us to hypothesise that this protein may act as a scaffold of the Hippo pathway. To test this hypothesis, we immunoprecipitated the endogenous proteins from cells grown in full media (10% serum) and, in order to trigger the activation of the Hippo pathway’s pro-apoptotic signal, we serum-deprived the cells (0.1% serum). Our results confirmed that IQGAP1 binds specifically to MST2 and LATS1 (Figure 1A,B). Additionally, we confirmed the interaction of IQGAP1 with YAP1; however, contrary to what was shown before by Sacks’s group, serum deprivation decreased the interaction of the endogenous proteins. Importantly, our data showed that the IQGAP1 interaction with MST2 and LATS1 clearly decreased upon serum deprivation too. We observed similar results when we performed similar experiments in HeLa cells (Figure 1D). Collectively, these results confirmed that IQGAP1 is a dynamic interactor of these proteins, indicating that this scaffold might be a regulator of the Hippo signalling network. 

### 3.2. IQGAP1 Scaffolds the MST2-LATS1 Interaction and Regulates Their Activation

**Figure 2:**
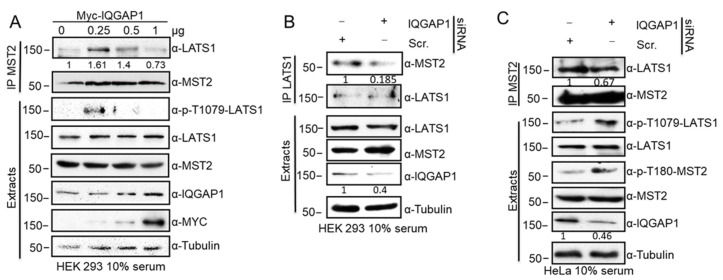
IQGAP1 scaffolds MST2-LATS1 interaction and regulates the activation of the kinases of the pathway. (**A**) HEK 293 cells were transfected with the indicated amounts of Myc-IQGAP1 constructs. Endogenous MST2 immunoprecipitates were analysed by western blot with the indicated antibodies. IP blots were quantified using ImageJ and the numbers show relative fold change of LATS1 normalised by MST2. (**B**) HEK 293 cells were transfected with IQGAP1 siRNA or a non-targeting (Scr.) siRNA pool. Endogenous MST2 and LATS1 immunoprecipitates were examined by western blotting. IQGAP1 and Tubulin blots were quantified using ImageJ and the numbers shows relative fold change of IQGAP1 normalised by Tubulin. IP blots were quantified using ImageJ and the numbers show relative fold change of MST2 normalised by LATS1. (**C**) HeLa cells were transfected with IQGAP1 siRNA or a non-targeting (Scr.) siRNA pool. Endogenous MST2 immunoprecipitates were examined by western blotting. Blots were quantified as in (**A**).

The observation that IQGAP1 interacts with the three core proteins of the Hippo pathway indicates that this protein may have a previously unknown scaffolding function at the core proteins of this pathway. To determine whether IQGAP1 functions as a classical scaffold [33,34] for the pathway, it was necessary to monitor how expression of the proteins altered the MST2-LATS1 complex. We observed that gradient overexpression of IQGAP1 in HEK 293 cells regulated the MST2-LATS1 interaction in a dose-dependent, non-linear manner by increasing complex formation at lower levels (0.25 and 0.5 µg) but disrupting it at higher concentrations (Figure 2A). Additionally, we observed a concomitant increase of LATS1 phosphorylation on T1079, which indicated that LATS1 kinase activity is regulated by IQGAP1, again in a concentration-dependent, non-linear manner (Figure 2A). Conversely, downregulation of IQGAP1 levels in HEK293 cells by transfecting a specific siRNA resulted in a reduction of MST2-LATS1 interaction (Figure 2B). This observation was confirmed in HeLa cells, where we also observed a decrease of MST2-LATS1 interaction upon IQGAP1 downregulation. Paradoxically, knocking down the expression of IQGAP1 increased the phosphorylation activating sites within LATS1 and MST2 (Figure 2C), indicating that IQGAP1 limits the activation of these kinases when expressed at endogenous levels. Altogether, this confirmed that IQGAP1 not only scaffolds the core kinases of the Hippo cascade but may also channel the signal mediated by these kinases.

### 3.3. MST2 and LATS1 Bind to the IQ Domain of IQGAP1

**Figure 3:**
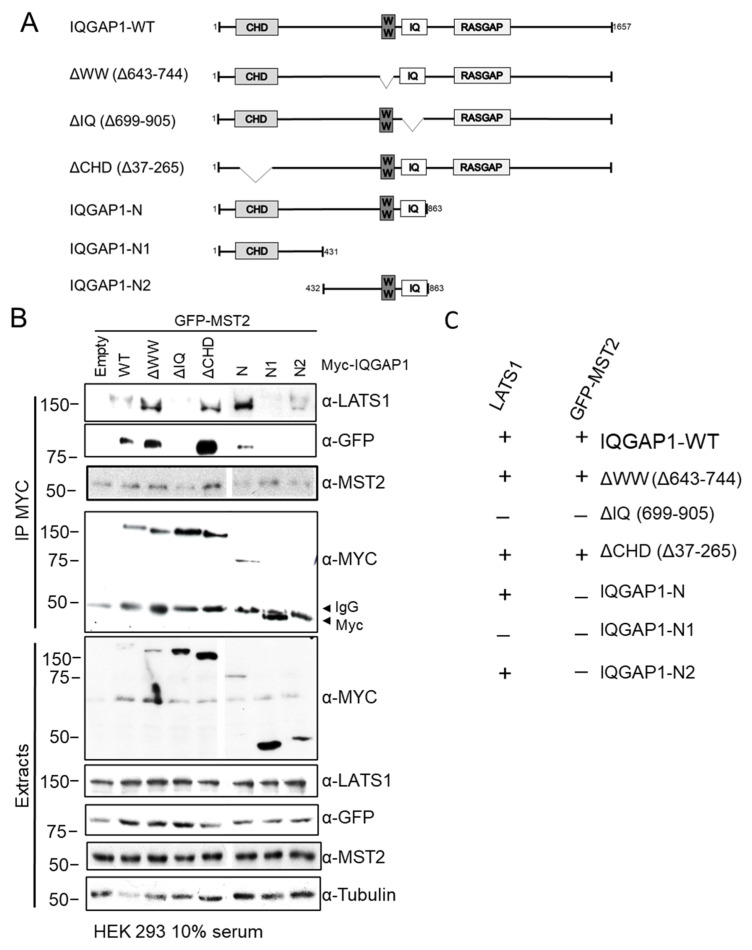
LATS1 and MST2 bind to IQGAP1 IQ domains. (**A**) A schematic representation of IQGAP1 full length and domain deletion mutants of IQGAP1. The protein interaction domains and the amino acid residues of each mutant are indicated. IQGAP1 Myc-tagged constructs used were wild-type IQGAP1 (WT), IQGAP1ΔWW (ΔWW), IQGAP1ΔIQ (ΔIQ), IQGAP1ΔCHD (ΔCHD), IQGAP1-N (N), IQGAP1-N1 (N1) or IQGAP1-N2 (N2). (**B**) HEK 293 cells were co-transfected with different Myc-IQGAP1 (2 µg) tagged deletion constructs and with GFP-MST2 (1 µg). Myc-IQGAP1 immunoprecipitates were analysed by western blotting with indicated antibodies. IgG heavy chain unspecific band and Myc specific band (~50 KDa) are indicated with ◄. All blots were spliced from the same blots and a gap has been left to make this clear. (**C**) Table summarises the ability of LATS1 and MST2 to bind (+) or not (−) to IQGAP1 fragments.

We next decided to map the protein–protein interaction domains required for the interaction between MST2 and LATS1 with IQGAP1. To do this, we used a series of IQGAP1 domain-deletion mutants tagged with the Myc-epitope previously generated by Sacks’s group (Figure 3A) [20,35]. These plasmids express IQGAP1 mutants devoid of the CHD, IQ and WW domains (ΔCHDβ, ΔIQ and ΔWW). Additionally, we used constructs expressing the N-terminal region of IQGAP1 (IQGAP1-N), two constructs that express partial regions of the N-terminal region (IQGAP1-N1 and IQGAP1-N2). The plasmids were transiently expressed in HEK293 cells and the interaction of MST2 and LATS1 was monitored by immunoprecipitating the panel of IQGAP1 mutants using Myc-tag antibody. Immunoprecipitation assays showed that LATS1 interacts with IQGAP1 via its IQ motif (summarised in Figure 3A), since no interaction between LATS1 and IQGAP1 ΔIQ was detectable. This was further confirmed by the observation that LATS1 did not interact with IQGAP1-N1, which also lacks the IQ motif. Of note, the results with the IQGAP1 ΔIQ also indicated that LATS1 does not interact with the C-terminal domains, since they are present in this construct and no interaction is shown. 

We also monitored the interaction of endogenous MST2 with these constructs. However, MST2 was immunoprecipitated in our control cells, which do not express Myc-tagged constructs (Figure 3B MST2 blot), which was surprising since we have demonstrated that the endogenous IQGAP1-MST2 proteins are specific binders (Figure 1B). This observation may be explained by an unspecific recognition of MST2 by Myc-tag antibody. Alternatively, this could be explained by previous data that indicate that members of the MST1-4 family may form a complex with Myc and regulate the phosphorylation of this transcription factor by NDR kinases (Cornils et al. 2011). Therefore, in order to map the MST2-IQGAP1 interaction, we decided to overexpress GFP-MST2 and immunoprecipitated the complexes using Myc antibody. Using this construct, we could see a specific immunoprecipitation of MST2-IQGAP1 complex and map the interacting domains mediation this interaction. The data showed that this kinase also interacts with the IQ domain of IQGAP1 (Figure 3B), demonstrating that both LATS1 and MST2 bind to IQGAP1 through the IQ domain. However, we did not see an interaction between GFP-MST2 and IQGAP1-N2, indicating that the IQ domains are necessary but not sufficient to mediate the interaction between IQGAP1 and MST2. 

### 3.4. MST2 and LATS1 Cooperate to Bind to IQGAP1

**Figure 4:**
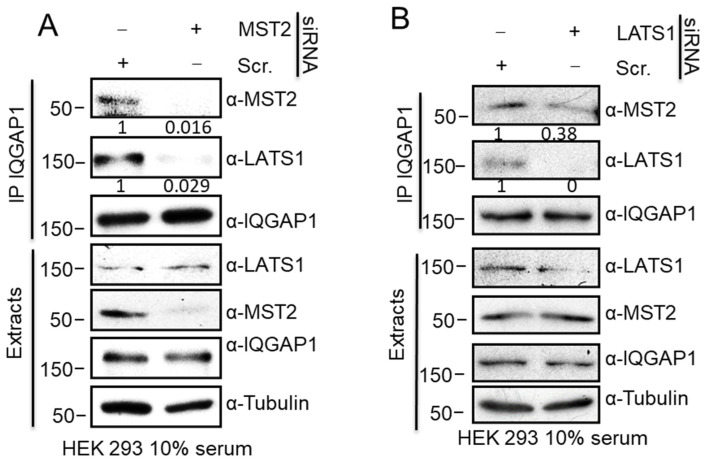
MST2 regulates LATS1 interaction with IQGAP1. (**A**). HEK 293 cells were transfected with MST2 siRNA or a non-targeting (Scr) siRNA pool. Cells were lysed after 48 h and IQGAP1 immunoprecipitates were examined by western blotting. (**B**) HEK 293 cells were transfected with LATS1 siRNA or a non-targeting siRNA pool. IQGAP1 immunoprecipitates were examined by western blotting. IP blots were quantified using ImageJ and the numbers show the relative fold change of MST2 or LATS1 normalised by IQGAP1 blots.

The IQ region of IQGAP1 is formed by the repetition of four IQ domains and has been shown to bind members of the MAPK pathway, i.e., RAF and ERK, simultaneously [25]. It is, therefore, plausible that MST2 and LATS1 bind simultaneously to IQGAP1 via the IQ motif. Alternatively, one of the proteins may prime the recruitment of the other by enhancing its binding to the IQGAP1 complex. A further possibility is that LATS1 and MST2 compete for the binding to this domain and exclude the alternative interaction to IQGAP1. The latter scenario is unlikely, since we saw an IQGAP1-dependent increase of LATS1 binding to MST2 (Figure 2A). Nevertheless, to test which of these scenarios was more likely, we decided to downregulate the expression of MST2 or LATS1 and examine whether there was any change in the complex formation between the other kinase and IQGAP1. The immunoprecipitation assays showed that the interaction between LATS1 and IQGAP1 was severely reduced when MST2 levels were downregulated using siRNA, indicating that MST2 facilitates the interaction of LATS1 and IQGAP1, further supporting the idea that both proteins do not compete for the interaction with IQGAP1 (Figure 4A). Similarly, we tested if LATS1 was necessary for the formation of an MST2-IQGAP1 complex by downregulating LATS1. The immunoprecipitation assay showed that the interaction between MST2 and IQGAP1 is only partially decreased by the downregulation of LATS1, indicating that MST2 would mainly bind to IQGAP1 independent of LATS1 (Figure 4B). Considering these results, we concluded that MST2 is crucial for priming the interaction between LATS1 and IQGAP1.

### 3.5. IQGAP1 Regulates MST2-LATS1-Dependent Apoptosis

**Figure 5:**
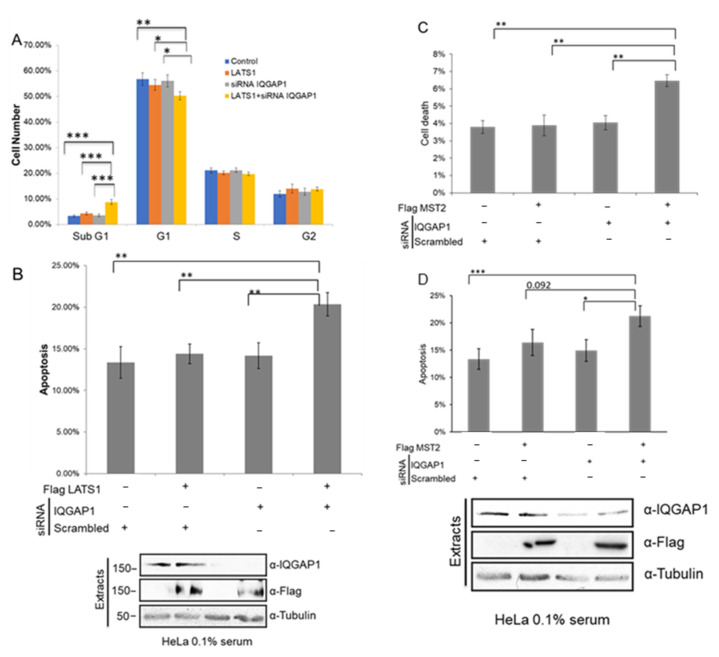
IQGAP1 regulates LATS1- and MST2-dependent apoptosis. (**A**) HeLa cells co-transfected with IQGAP1 or non-targeting siRNA pool and Flag-LATS1 and serum deprived for 16 h. Cell cycle distribution was assessed by PI staining using flow cytometry. (**B**) Upper panel: Caspase 3/7 activation of HeLa cells transfected as in A measured by FITC-VAD-FMK binding after starvation. Lower panel: Total lysates corresponding to the apoptotic assay analysed by western blot. (**C**) HeLa cells co-transfected with IQGAP1 or non-targeting siRNA pool and Flag-MST2 and starved for 16 h. Cell death was assessed by PI staining using flow cytometry and measuring subG1 population. (**D**) Upper panel: Caspase activity of HeLa cells transfected as in C measured by FITC-VAD-FMK binding after starvation. Lower panel: Total lysates corresponding to the apoptotic assay analysed by western blot. *p*-values were obtained by Student’s *t*-test, *n* = 3, error bars indicate SEM, * = *p* < 0.05, ** = *p* < 0.01, *** = *p* < 0.001.

The above-explained data strongly indicated that IQGAP1 scaffolds the MST2-LATS1 complex. Importantly, IQGAP1 also seems to regulate the activation of both kinases, and our downregulation experiments indicated that IQGAP1 expression supresses kinase activation. Hence, we wanted to test whether these biochemical and mechanistic observations correlated with a regulation of specific cellular functions. Both IQGAP1 and LATS1 have been shown to regulate cell cycle progression. LATS1 overexpression has previously been shown to increase the rate of apoptosis and result in G2/M cell cycle arrest [36]. Similarly, IQGAP1 has been identified as a regulator of the cell cycle and translocates to the nucleus in late G1 phase triggering DNA replication [19]. For these reasons, we posit that IQGAP1 may regulate the role of MST2 and LATS1 in the cell cycle. Therefore, we decided to test this hypothesis by monitoring the effect that concomitant overexpression of the Hippo kinases and downregulation of IQGAP1 has in cell cycle progression. The cell cycle profile showed no significant changes between S-phase and G2-phase in any of the conditions (Figure 5A). Importantly, while suboptimal overexpression of LATS1 or downregulation of IQGAP1 had very little effect on the percentage of cells in the G1-phase, concomitant overexpression of LATS1 and downregulation of IQGAP1 resulted in a decrease of the number of cells in this phase of the cell cycle. Since we did not observe an accumulation of cells in S-phase or G2/M-phase, but we saw a substantial increase of cells in the sub-G1 population (Figure 5A), we hypothesised that this change of cells in G1-phase was not due to cell cycle arrest but to an increase in cell death. 

To test if this increase of cell death was due to apoptosis, activation of caspase 3/7 was measured by flow cytometry (Figure 5B). When we transfected suboptimal concentrations of LATS1 or downregulated IQGAP1 levels using siRNA, we did not observe significant changes in the level of apoptosis in these cells. However, the concomitant expression of LATS1 and downregulation of IQGAP1 resulted in a significant increase in apoptosis indicating that IQGAP1 prevents LATS1-dependent apoptosis in these cells. Considering these data, we also wanted to test whether MST2-dependent apoptosis was negatively regulated by IQGAP1. To do so, we overexpressed MST2 and/or downregulated IQGAP1 in HeLa cells. Concomitant overexpression of MST2 and downregulation of IQGAP1 resulted in an increase of apoptosis (Figure 5C,D). These data strongly indicate that IQGAP1 is a negative regulator of the MST2-LATS1 pro-apoptotic signal. 

### 3.6. IQGAP1 Regulates YAP-p73 Interaction and Transcriptional Activity

**Figure 6:**
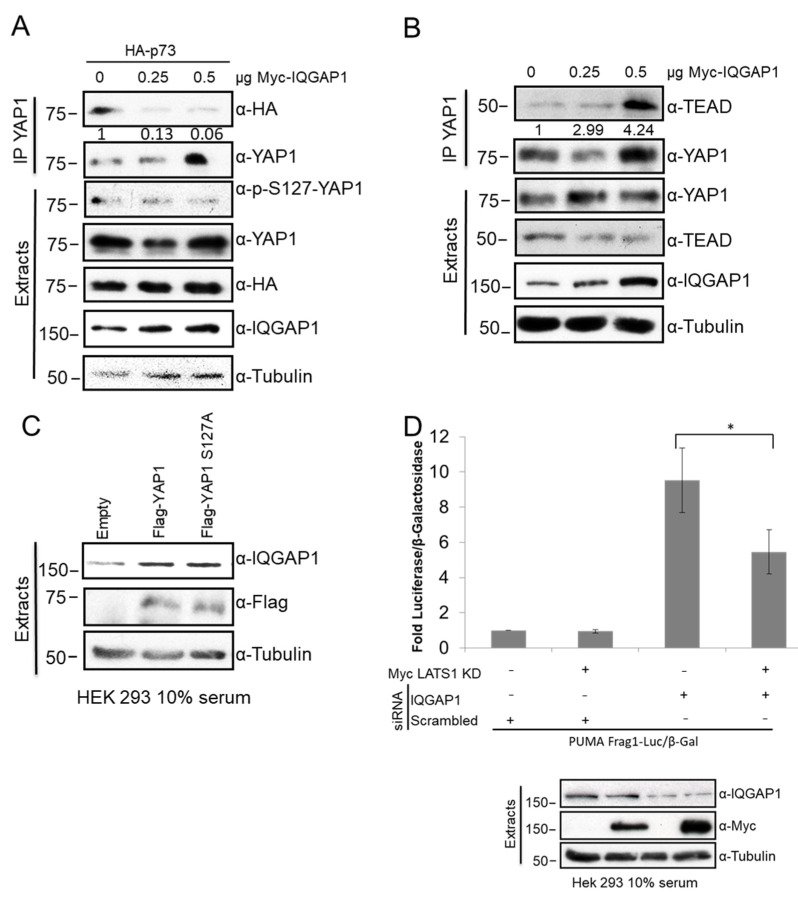
IQGAP1 YAP interactions and YAP-dependent transcription. (**A**)** YAP1 immunoprecipitates from HEK 293 cells co-transfected with the indicated amounts of Myc-IQGAP1 and HA-p73 (1 µg) constructs. HA-p73 co-immunoprecipitation levels were measured by western blot. IP blots were quantified using ImageJ and the numbers show relative fold change of MST2 or LATS1 normalised by IQGAP1 blots. (**B**)** YAP1 immunoprecipitates from HEK 293 cells co-transfected with the indicated amounts of Myc-IQGAP1 construct. TEAD co-immunoprecipitation levels were measured by western blot. (**C**) Total protein extracts from HEK 293 cells transfected with Flag-YAP1, Flag-YAP1-S127A or the corresponding empty vector analysed by western blot. (**D**) Upper panel: Luciferase assay of PUMA promoter activity in HEK 293 cells co-transfected with PUMA Frag1-Luc and β-Gal plasmids, and LATS1 kinase dead mutant (KD) or the corresponding empty vector and IQGAP1 siRNA or the corresponding non-targeting siRNA pool. Luciferase activity normalised against β-galactosidase signal. Lower panel: Total lysates corresponding to the luciferase assay measured by western blot. *p*-values were obtained by Student’s *t*-test, *n* = 3, error bars indicate SEM, * = *p* < 0.05.

The observation that IQGAP1 regulates MST2-LATS1-dependent apoptosis and that YAP1 is also an IQGAP1 interactor led us to test next the possible role of this scaffold as regulator of YAP1-dependent transcription downstream of MST2 and LATS1 signalling. We first monitored if IQGAP1 regulates YAP1-p73 interaction. To do this, we transfected increasing amounts of IQGAP1 in HEK 293 cells and performed immunoprecipitation of endogenous YAP1. We observed that overexpression of IQGAP1 disrupts the YAP1-p73 complex (Figure 6A). We have previously shown that YAP1 pro-apoptotic signal requires LATS1 phosphorylation of YAP1, loss of YAP1-LATS1 interaction and the increase of p73-YAP1 complex [5]. Thus, our data are in agreement with the idea that IQGAP1 prevents the Hippo pro-apoptotic pathway. Importantly, we also examined the effect of IQGAP1 overexpression in YAP1 phosphorylation status and we detected no changes of YAP1-S127 phosphorylation (Figure 6A). This result indicated that the effect of IQGAP1 on YAP1-p73 interaction is independent of YAP1-S127 phosphorylation status. Furthermore, increasing amounts of transfected IQGAP1 in HEK 293 cells promoted the pro-growth YAP1-TEAD interaction, although it also induces a clear decrease of TEAD expression levels (Figure 6B). Altogether, these data indicate that IQGAP1 impairs the formation of the YAP1-p73 pro-apoptotic complex and regulates the YAP1-TEAD complex.

Next, we decided to test if YAP1 can regulate IQGAP1 expression levels. Previous data have shown that bile acid treatment in hepatocytes results in an overexpression of IQGAP1 that in turn can regulate YAP1 transcriptional activity [21]. We hypothesised that this increase of IQGAP1 expression might be mediated by YAP1. To test this, we decided to overexpress YAP1 and the mutant YAP1-S127A and monitor the effect that this has on the level of expression of IQGAP1. The data showed that there was a similar level of increase of endogenous IQGAP1 expression upon overexpression of both YAP proteins (Figure 6C). Thus, these data indicate that YAP1 regulates IQGAP1 protein levels.

Our data showed that IQGAP1 negatively regulates the YAP1-p73 interaction and that loss of expression of IQGAP1 in combination with suboptimal expression of LATS1 results in the activation of apoptosis. Moreover, our published data have shown that LATS1-dependent activation of YAP-p73 transcriptional activity results in the expression of the pro-apoptotic protein PUMA [5]. Thus, we hypothesised that IQGAP1 may regulate LATS1-dependent transcription of PUMA. To test this, we transfected HEK 293 cells with a p73 luciferase reporter that contains the p73 responsive elements of the PUMA gene promotor and downregulated IQGAP1 expression using specific siRNAs. Additionally, in order to demonstrate that any possible effect of IQGAP1 in p73-dependent transcription is mediated by the Hippo pathway, we decided to inhibit the pathway by expressing a kinase dead mutant LATS1 that behaves as a dominant inhibitory of endogenous LATS1. The experiment showed that overexpression of LATS1 KD has a non-significant effect on PUMA transcription, but the knock down of IQGAP1 resulted in a seven-fold increase of luciferase activity (Figure 6D). Interestingly, concomitant downregulation of IQGAP1 protein levels and expression of LATS1 KD rescued the effect of IQGAP1 in p73 transcriptional activity and resulted in a three-fold decrease of luciferase activity. These data clearly indicate that LATS1 kinase activity is necessary for the activation of p73 caused by IQGAP1 downregulation. Altogether, these experiments indicated that IQGAP1 is a negative regulator of YAP1-p73 activity and further supported the idea that this scaffold prevents the activation of apoptosis through the negative regulation of the core proteins of the Hippo pathway.

### 3.7. The IQGAP1-Hippo Module Is Regulated by CDCA in Hepatocellular Cells

**Figure 7:**
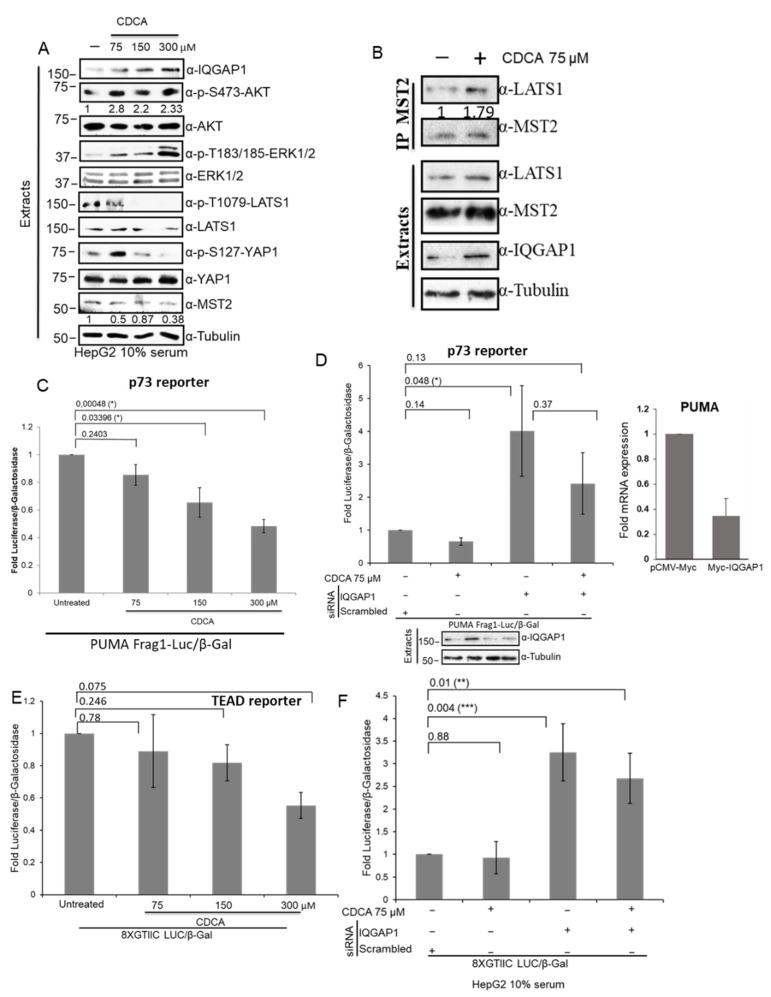
CDCA overload induces IQGAP1 expression and deactivates Hippo signalling. (**A**) HepG2 cells were treated with increasing concentrations of CDCA (75, 150 or 300 µM) for 8 h and the cells were lysed. Cell extracts were analysed by western blotting with indicated antibodies. Blots were quantified using ImageJ and the numbers show relative fold change of AKT phosphorylation normalised by total levels of AKT and level of expression of MST2 normalised by tubulin levels. (**B**) HepG2 cells were treated with 75 µM CDCA for 8 h. MST2 was immunoprecipitated from cell extracts using specific antibody. Immunoprecipitated proteins and cell extracts were blotted with the indicated antibodies. IP blots were quantified using ImageJ and the numbers show relative fold change of LATS1 normalised by MST2 blot. (**C**) HepG2 cells were co-transfected with PUMA promotor luciferase reporter (PUMA Frag1-Luc, p73 reporter) and β-galactosidase construct. Forty-eight hours after transfection, the cells were treated for 8 h with the indicated concentrations of CDCA. p73 transcriptional activity was measured by luminescence and β-galactosidase enzymatic activity was measured by absorbance. (**D**) Left panel. HepG2 cells were co-transfected with PUMA and β-galactosidase reporters and 50 ng/mL IQGAP1 or non- targeting siRNA pool where indicated. Forty-eight hours after transfection, the cell were treated for 8 h with 75 μM CDCA. Transcriptional activity was measured as in (**C**). Lower panel shows protein expression of the indicated proteins determined by western blot in a representative experiment. Right panel. HepG2 cell were transfected with 0.5 μg Myc-IQGAP1 or pCMV-Myc plasmids and PUMA mRNA expression was measured by rtPCR. The graph shows PUMA mRNA levels normalised by GAPDH mRNA expression. (**E**) HepG2 cells were transfected with 8XGTIIC LUC construct (TEAD reporter) β-galactosidase plasmid. Forty-eight hours after transfection, the cells were treated for 8 h with increasing concentrations of CDCA. TEAD transcriptional activity was measured and β-galactosidase enzymatic activity were measured as in C. (**F**) HepG2 cells were co-transfected with 8XGTIIC LUC construct (TEAD reporter) β-galactosidase plasmid and 50 ng/mL IQGAP1 or non-targeting siRNA pool, where indicated. Forty-eight hours after transfection, the cells were treated for 8 h with 75 μM CDCA. After lysis, TEAD transcriptional activity was determined as in (**C**). *p*-values were obtained by Student’s *t*-test, *n* = 3, error bars indicate mean SEM. * = *p* < 0.05, ** = *p* < 0.01, *** = *p* < 0.001.

IQGAP1 mRNA/protein levels are upregulated in hepatocellular carcinoma (HCC) [37], a cancer type where loss of the core proteins of the Hippo pathway and upregulation of YAP1 proliferative signal have been shown to result in cancer development [1]. Importantly, Anakk et al. showed a connection between the Hippo pathway and deregulation of IQGAP1. In particular, this work showed that bile acid treatment increased IQGAP1 expression levels and that MST2, LATS1 and YAP were deregulated [21]. Considering all these data and the results that we have presented so far, we hypothesised that IQGAP1 may play a role in the deregulation of the core components of the Hippo pathway in HCC. To test this, we decided to use the HCC cell line HepG2, which has been shown to respond to bile acid treatment [38]. We first monitored the changes that treatment with bile acid causes in the proteins of the IQGAP1-Hippo module by treating the cells with increasing concentrations of chenodeoxycholic acid (CDCA) for 8 h. We saw an increase in the level of protein expression of IQGAP1 that is directly related to the concentration of CDCA (Figure 7A). Interestingly, the level of activation of LATS1 was decreased by treatment with CDCA, and in fact, no phosphorylation of the activating LATS1-T1079 was detected when the cells were treated with higher concentrations of CDCA. It is worth noting that the levels of expression of MST2 were reduced when the cells were treated with CDCA. On the other hand, no significant effect of CDCA treatments was shown for YAP1 protein levels. Intriguingly, we saw an increase of YAP S127 phosphorylation when the cells were treated with low concentration of CDCA (75 µM), but a decrease on the phosphorylation of this residue was observed when the cells were treated with the highest concentration. Additionally, we saw and increase of LATS1-MST2 interaction in response to low concentrations of CDCA treatment (Figure 7B). Importantly, we also saw a concomitant activating phosphorylation of AKT (Figure 7A), a negative regulator of MST2 kinase activity [4,26], which may explain the lack of activation of LATS1, even though there is an increase of interaction of MST2-LATS1 complex. Finally, our data show that CDCA causes a concomitant activation of the ERK pathway. These results indicated that CDCA might lead to cellular transformation by concurrently shutting down MST2 pro-apoptotic signal and activating the tumorigenic MAPK and AKT pathways by increasing the expression of IQGAP1.

To confirm if CDCA regulation of IQGAP1 has a negative effect in the Hippo-dependent apoptotic signal, we next tested the effect of this treatment on p73-dependent transcription. Using a luciferase reporter gene under the control of the promoter sequence of PUMA, we observed that p73 transcriptional activity was inversely correlated with the concentration of CDCA treatment (Figure 7C). To test if this was dependent on IQGAP1 signalling, we downregulated the IQGAP1 expression levels using specific siRNA. Similar to what we saw in HEK 293 cells, downregulation of IQGAP1 expression seems to cause an increase of PUMA transcription (Figure 7B). This increase of PUMA transcription is partially rescued by treatment with low concentrations of CDCA, which may be mediated by the remaining molecules of IQGAP1. Nevertheless, these data indicate that the treatment with bile acids inhibit the Hippo pathway pro-apoptotic activity. To further confirm that IQGAP1 regulates PUMA transcription, we overexpressed this protein and measured PUMA mRNA expression by rtPCR (Figure 7D, right panel). This experiment shows that an increase of IQGAP1 protein levels decreases the transcription of the PUMA gene in this cell line. Since we saw that CDCA treatment caused a reduction of the YAP1-p73 transcription target PUMA, we tested if this bile acid also regulated the transcriptional activity of TEAD, as would be expected by the canonical view of the pathway [2,39]. Interestingly, our data, generated using TEAD luciferase reporter, indicated that high CDCA concentrations cause a reduction of TEAD transcriptional activity, which would show that the proliferative effect mediated by YAP1 upon bile acid stimulation is not mediated by this transcription factor (Figure 7D). Moreover, we saw that there seems to be an increase of TEAD-dependent transcription when we downregulated the expression of IQGAP1, indicating that this protein negatively regulates TEAD transcription in this cell line. These data indicated that CDCA treatment prevents YAP1-TEAD-dependent transcription. Remarkably, this result is in line with the observation from Sacks’s group that showed that IQGAP1 prevented YAP1-TEAD-dependent transcription, supporting that this scaffold negatively regulates the pro-survival transcription mediated by this complex.

## 4. Discussion

Our study shows that IQGAP1 is a regulator of the non-canonical Hippo signalling network. The detailed characterisation of the biochemical changes mediated by IQGAP1 on the MST/Hippo pathway allows us to obtain a working model of the mechanistic regulation of these complexes. Altogether, the results indicate that IQGAP1 increases the interaction between LATS1 and MST2 when it is expressed at optimal concentrations but higher or lower concentrations of IQGAP1 reduce the formation of the MST2-LATS1 complex. This clearly resembles the bell-shaped curve effect that changes in scaffold protein concentrations have in the protein interactions that they regulate [34,40,41] and indicates that IQGAP1 is a scaffold of the pathway. Further support for this finding comes from the observation that IQGAP1 also regulates the MST2 and LATS1 kinase activity in a concentration-dependent manner. Interestingly, despite the promotion of the formation of LATS1-MST2 complex, IQGAP1 seems to be an inhibitor of MST2 and LATS1 kinase activity, since we observed a decrease of the phosphorylation levels of these kinases when the interaction is promoted by IQGAP1. Conversely, downregulation of IQGAP1 expression results in an increase of MST2 and LATS1 phosphorylation further confirming that IQGAP1 inhibit these kinases. Our results also indicate that IQGAP1 regulation of the Hippo pathway might be cell type-specific since MST2 and LATS1 activation showed clear differences between HeLa and HEK293 cells. For instance, the increase of activation caused by IQGAP1 depletion is not due to an increase of LATS1-MST2 interaction in HeLa cells while there is an increase of this complex in HEK293 cells. A possible explanation for this difference is that MST2 and LATS1 may interact with other activating proteins when they are released from IQGAP1 inhibitory binding. Furthermore, the activation of LATS1 and MST2 upon release from IQGAP1 inhibitory binding might also be independent of each other in some cell types, as previously described [1]. Finally, our data indicate that the inhibition of MST2 and LATS1 kinases activity by IQGAP1 seems to be directly related to the regulation of the pro-apoptotic signal mediated by this kinase cassette, potentially through YAP1.

Importantly, in the current study, we have also characterised in detail how IQGAP1 interacts with MST2 and LATS1. As with most classical scaffolds, IQGAP1 is a multidomain protein that mediates its effects in signal transduction by protein–protein interactions with its effectors [41]. Using IQGAP1 deletion mutants generated by Sack’s group, we have mapped that MST2 and LATS1 bind to the IQ domains of IQGAP1, the same domains that mediate YAP1 interaction with this scaffold [20]. IQGAP1 has four IQ domains and our data indicate that LATS1 and MST2 bind to these domains at the same time. Importantly, despite MST2 and LATS1 binding to the IQ domains, our experiments show that they do not compete for the interaction with IQGAP1. This is probably because they bind to different IQ domains. Interestingly, our results show that while MST2 binding to IQGAP1 is not affected by LATS1, LATS1 binding to IQGAP1 requires the previous binding of MST2 to the scaffold, indicating that MST2 promotes LATS1 binding to IQGAP1. Remarkably, this is similar to what has been observed for RAF and MEK, which also bind to the IQ domains [17]. Thus, the IQ domains of IQGAP1 seem to mediate the interaction of different kinase cassettes. Of note, the observation that LATS1, YAP and MST2 bind to the same domains of IQGAP1 where RAF and MEK bind indicates that this scaffold might be important for the regulation of the crosstalk between these two pathways [1,4,9,42,43], which should be further explore in the future. 

Functionally, the results of our study indicate that IQGAP1 is an important regulator of the Hippo pathway biological effects. IQGAP1 seems to be a key regulator of MST2 and LATS1-dependent regulation of cell cycle that negatively regulates the pro-apoptotic signal mediated by these kinases. Moreover, IQGAP1 also causes the inhibition of the binding of YAP1 and p73 and their transcriptional activity of this complex. Our results also indicate that IQGAP1 prevents YAP1-TEAD-dependent transcription supporting the findings from Sacks’s group, which are complementary to our work [20]. In this previous study, the authors showed that IQGAP1 interacts with YAP1 and prevents the formation of YAP1-TEAD complexes in the nucleus causing a reduction of TEAD target genes. It must be noted that we can see an increase of YAP1-TEAD interaction in HEK293 cells when we overexpress IQGAP1. This would indicate that IQGAP1 does not prevent the formation of this complex, which was one of the possible hypotheses proposed by Sacks’s group to explain the repression of TEAD transcription. However, we can see a clear reduction of TEAD expression when IQGAP1 is overexpressed, supporting the idea that the repression is caused by loss of this transcription factor. Hence, IQGAP1-medited inhibition of YAP1 pro-apoptotic function cannot be explained by the canonical hippo pathway where inhibition of MST2 and LATS1 results in YAP1 “activation” due to lack of phosphorylation of the residue S127 [2]. This is further supported by the observation by both groups that changes of IQGAP1 levels do not regulate YAP1 phosphorylation as expected by the On–Off model of the canonical Hippo pathway. Second, we see a decrease of TEAD-YAP1-dependent transcription in HepG2 cells that can be explained by the mechanisms described by Sacks’s group. Third, the data show that the loss of expression of IQGAP1 in liver cancer cells results in an increase of LATS1 activation and concomitant increase of p73 and TEAD transcriptional activity, indicating that in this scenario LATS1 activation may trigger TEAD transcriptional activity in a non-canonical fashion. This finding is not really surprising in light of recent work from several groups which further indicate that the current dogma for the regulation of YAP1 is very simplistic and other kinases and phospho residues are important for the mediation of the signalling of this co-transcription factors [1,44,45]. Of note, in the current study we did not check if MST1, LAST2 and TAZ, the homologues of the core proteins of the Hippo pathway, also interact with IQGAP1, but given the similarities of the protein interaction domains of the different isoforms, this could be expected. 

Our results also help to shed light on the physiological relevance of IQGAP1 in cancer. In particular, our work may explain, at least in part, the effect that IQGAP1 has in liver cancer, where this protein is commonly overexpressed and is proposed to behave as an oncogene [46]. A previous study had already shown that IQGAP1 may promote liver cancer through YAP1 signalling [21]. This study showed that, in an animal model, bile acids treatment leads to the accumulation of IQGAP1 in liver cells and increases YAP1 proliferative signals, promoting liver carcinogenesis [21]. Here, we have confirmed this observation using HCC cells and, although follow up studies will be necessary, our results potentially start delineating the possible mechanisms that explain the previous in vivo observations. As shown before by Anakk et al., we observe that CDCA treatment increases the level of expression of IQGAP1 leading to a decrease of MST2 expression and of LATS1 activation. This decrease of MST2 expression may contribute to HCC development as indicated by the observation that MST1/2 knockout mice develop liver cancer [47], which stress the potential physiological relevance of the current findings. Importantly, as mentioned above, the decreased expression of IQGAP1 seems to cause an increase of p73 and TEAD transcriptional activity in HCC cells, while increased expression of this scaffold prevents YAP1-p73 interaction. Thus, increase expression of IQGAP1 in HCC could prevent the activation of the pro-apoptotic transcription mediated by this complex. Intriguingly, we also observed a concomitant increase of ERK and AKT activation and inhibition of LATS1 in cells treated with CDCA where we see an increase of IQGAP1 expression. These results are in line with previous observations showing that IQGAP1-dependent cell proliferation in HepG2 cells is mediated by AKT/ERK-dependent cell proliferation [35,46]. It must be noted that cholestasis is a marker of hepatocellular carcinoma resulting in an increase of bile acids in the liver and our work is potentially related to this effect [21]. In vivo observations clearly support the importance of bile acids regulation of Hippo signalling in liver cancer. Thus, Zhou’s group showed that FGFR4 activation by FGF15 leads to a prevention of the inhibitory binding of RAF1 and MST1/2 and subsequent activation of the Hippo pathway in mice [23]. In this work, it was also shown that bile acid depletion delays liver tumorigenesis driven by MST1/2 depletion. In light of all this evidence, we postulate that pathological accumulation of CDCA may inhibit both the pro-apoptotic signal of the Hippo pathway by promoting the over-expression of IQGAP1. In turn, pathological over-expression of IQGAP1 would promote transformation by activating the pro-survival signals mediated by the MAPK and AKT pathway. Hence, it is tempting to speculate that the induction of liver cancer caused by cholestasis may require the loss of IQGAP1-Hippo pathway signalling and the activation of proliferative signals mediated by other pathways. Therefore, IQGAP1 seems to be a key regulator of a complex signalling network formed by the EKR, AKT and Hippo pathways. Remarkably, deregulation of IQGAP2 and IQGGAP3 have also been associated with liver cancer. IQGAP2 has been proposed to be a tumour suppressor and its expression is downregulated when IQGAP1 is overexpressed in hepatocellular [37,48,49] carcinoma, while IQGAP3 shows oncogenic properties. These proteins could potentially regulate the Hippo pathway and have different effect in the output of the complex role of the Hippo pathway in HCC.

In summary, we have shown that IQGAP1 is the scaffold of the core proteins of the Hippo pathway, which is likely to mediate the complex signalling network form by this pathway and the AKT and ERK pathways. Although further work is necessary to fully characterise this complex network, the IQGAP1-Hippo module could potentially be targeted for cancer therapy, especially in HCC. 

## Data Availability

The published proteomics data mentioned in this study [10,31,32], are available in the mass spectrometry proteomics data have been deposited to the ProteomeXchange Consortium [50], via the PRIDE partner repository identifier PXD018903 or upon request.
